# Carbopol 934-Sodium Alginate-Gelatin Mucoadhesive Ondansetron Tablets for Buccal Delivery: Effect of pH Modifiers

**DOI:** 10.4103/0250-474X.73912

**Published:** 2010

**Authors:** N. R. Kotagale, C. J. Patel, A. P. Parkhe, H. M. Khandelwal, J. B. Taksande, M. J. Umekar

**Affiliations:** Smt. Kishoritai Bhoyar College of Pharmacy, Behind Railway Station, New Kamptee, Dist. Nagpur, Maharashtra - 441 002, India

**Keywords:** Bioadhesion, buccal delivery, microenvironmental pH, Ondansetron hydrochloride

## Abstract

The present work aims at developing mucoahesive tablets of ondansetron hydrochloride using bioadhesive polymers like carbopol-934, sodium alginate and gelatin. Tablets prepared by direct compression using different polymer with varying ratio were evaluated for hardness, friability, uniformity of weight, disintegration time, microenvironmental pH, bioadhesion and *in vitro* release. Hardness, friability disintegration time and drug release were found within pharmacopoeial limit. Microenvironmental pH decreased whereas bioadhesive strength, water uptake, and *in vitro* release increased with increase in carbopol-934. Increasing sodium alginate and gelatin increased the microenviromental pH and decreased bioadhesive strength, water uptake and *in vitro* release. With a view to investigate the modulation of drug release from formulation by addition of pH modifiers viz. citric acid and sodium bicarbonate, the tablets with carbopol-934 (2.0), sodium alginate (0.5) and gelatin (6.5) were used and the effect of pH modifiers on microenvironmental pH, bioadhesion, water uptake, *in vitro* permeation and *in vitro* release was studied. Microenvironmental pH, bioadhesive strength, water uptake, *in vitro* release and permeation decreased with increasing concentration of citric acid whereas microenvironmental pH, water uptake and release were enhanced and bioadhesive strength was lowered with increase in sodium bicarbonate. Present study demonstrates carbopol-934, sodium alginate, gelatin polymer system with added pH modifier can be successfully formulated for buccal delivery of ondansetron with desired release profile.

An increasing interest in the development of novel mucoadhesive buccal dosage form meant for systemic delivery as well as local targeting have been seen in recent years. Buccal delivery leading to transmucosal absorption of a drug into the systemic circulation offers a number of advantages for drugs that suffer from extensive first pass metabolism and poor bioavailability. Higher bioavailability, administration of lower doses, avoidance of liver and/or gastrointestinal metabolism and irritation of the gastrointestinal membrane, high permeability due to rich blood supply, reduced risk of overdose, non-invasive administration, convenience of self-medication, improved patient compliance, feasibility of beneficial adjunct product to an existing product and reduced risk of infectious disease makes buccal mucosa an attractive alternative route for systemic delivery of drugs[1,2]. The pH of the medium and pKa of the drug may affect the release profile[3]. Weakly basic drugs like ondansetron hydrochloride with pH-dependent solubility can experience problem on release from controlled release dosage forms[4]. Penetration of fluid with higher pH may cause conversion of more ionizable drug to a less soluble base and therefore diffusion of the drug from the matrix. Because of which, formulation of weakly basic drugs for oral administration can be expected to result in particularly variable release rates with changes in pH of the surrounding fluids[5]. The objective of transmucosal formulation designs for weakly basic drug is enhancing the bioavailability and reduce variability. Polymeric film coating is frequently used to control drug release from solid pharmaceutical dosage forms[6–10]. An anionic polymer, sodium alginate has been reported to produce pH-independent release of a basic drug, verapamil HCl from a hydrophilic HPMC-based matrix tablet where sodium alginate has altered the pH in the matrix tablets[11]. The permeability of a non-ionized drug is likely to increase across an epithelial barrier, and this may be achieved by a change in pH of the drug delivery system[12–14]. Absorption of some drugs via the buccal mucosa is found to increase when carrier pH is lowered[4]. Increasing levels of pH modifiers progressively enhance drug release. The incorporation of pH modifiers such as citric, fumaric or sorbic acid is a common approach employed with matrix and coated systems[15–18], but not very common with buccal adhesive drug delivery systems[19]. Release of weakly basic drug from swellable tablets prepared with hydrophilic polymers was enhanced by incorporating pH modifiers, such as succinic, fumaric or adipic acid[18,20,21]. The addition of fumaric acid to drug/alginate-based matrix systems have decreased the microenvironmental pH within the tablets and resulted into increase in the solubility of the weakly basic drug at higher pH[4].

Ondansetron is a serotonin (5-hydroxytryptamine) subtype 3 (5-HT_3_) receptor antagonist used in the management of nausea and vomiting associated with cancer chemotherapy, radiotherapy and surgery. Ondansetron has been reported to produce adverse events such as, headache, constipation and diarrhea, which are mild to moderate and rarely require treatment. Following oral administration, it is absorbed rapidly but extensively metabolized by liver. Antiemetic drugs tend to be discharged by vomiting[22], but on the contrary, intravenous administration renders rapid effects to a patient, but the onset is too rapid to cause undesirable effects with local pain[23].

Therefore, in an attempt to prepare buccal mucoadhesive formulations for rapid delivery devoid of first pass metabolism, this study was undertaken to formulate a buccal mucoadhesive drug delivery system for ondensetron. Investigations were further directed to evaluate the effect of pH modifiers, citric acid (CA) and sodium bicarbonate (SB), on *in vitro* permeation, drug release and formulation properties.

## MATERIALS AND METHODS

Ondansetron HCl was received as a generous gift from commercial suppliers (Ellis Pharma Pvt. Ltd., Ahmedabad, India). Carbopol-934 (C), gelatin (G), sodium alginate (SA), magnesium stearate, microcrystalline cellulose (Loba Chemie Laboratory Chemicals Ltd., India); citric acid, sodium bicarbonate (RFCL Fine Chemicals Ltd., India) used were of either analytical or pharmaceutical grade.

### Tablet preparation:

The three polymers Carbopol-934, sodium alginate and gelatin (C:SA:G) were used in combinations to prepare bioadhesive tablets of ondensetron HCl by direct compression method using a 10 station rotary tableting machine (Rimek Minipress-I, India). All the ingredients were sieved before use (No. 200, 75 μm). Accurately weighed quantities of drug (8 mg/tablet), polymer (varying drug: polymer ratio from 1:1 to 1:5) and diluent microcrystalline cellulose with added magnesium stearate (2%) were mixed and compressed, using 6.0 mm standard concave punches keeping the weight of tablet constant to 100 mg. Prepared tablets were evaluated for weight and content uniformity, hardness, thickness, friability and disintegration characteristics as per pharmacopoeial (IP-96/ USP-24) specifications. In addition to the above properties prepared tablets, formulations were also evaluated for microenvironmental pH, *in vitro* release, bioadhesion, water uptake and *in vitro* permeation.

### Microenvironmental pH:

The release of the drug exhibiting pH-dependent solubility is largely dependent upon the pH of the microenvironment[24] and release of weakly basic compound has been enhanced by incorporating adipic acid and fumaric acid[16,18,21,25]. Microenvironmental pH of tablets was determined by allowing the tablets to swell for 2 h in 4 ml distilled water (pH 6.5±0.05) in a fabricated glass tube. A pH-electrode was kept in contact with the tablet surface, equilibrated for 1 min and microenvironmental pH was determined by potentiometry[26].

### *in vitro* bioadhesion test:

Weight required for detachment of tablets from the porcine buccal mucosa was determined by using a bioadhesion test assembly[27]. Fresh porcine buccal mucosa obtained from the slaughter house was freed from underlying fat and tissue, washed with distilled water and then with phosphate buffer 6.8 at 37^°^. Mucosa cut into pieces was placed in phosphate buffer pH 6.8 for 5 min. Each individual piece was tied to a previously balanced (with 5 g) teflon block keeping mucosal side upward which is then lowered into the beaker containing phosphate buffer pH 6.8 maintained at 37±0.5. Tablets under test were moistened and stuck to the hanging cylinder on left hand side. The balance beam was raised with the removal of 5 g weight from right pan lowering the teflon cylinder along with the tablet with a force of 5 g and kept in this position for 3 min. The weight was increased gradually, till the tablet separated from the mucosa. The weights added in the right pan represent the bioadhesive force required to separate from mucosa.

### *in vitro* water uptake studies:

Water uptake of tablets of each formulation was evaluated using a 1% w/v agar gel plate[28,29]. Twenty-four tablets divided into six groups, each group consisting of four tablets were weighed and average weight of four tablets was calculated. These tablets were placed on the gel surface in six Petri dishes, each containing four tablets and kept in an incubator at 37±1^°^. Each Petri dish was removed at one-hour interval for 6 h. The excess water on the surface of each tablet was blotted using a Whatman filter paper and the swollen tablets in each Petri dish were weighed. The average weight of the swollen tablets was calculated. Water uptake was calculated using the formula[30,31], Water uptake (g) = (W_1_-W_2_)/W_1_, where, W_2_ is the average weight of four tablets and W_1_ is the average weight of the swollen tablets

### *in vitro* release studies:

*in vitro* drug release was determined by USP method-II at a temperature of 37±1^°^and paddle speed of 50 rpm using 500 ml phosphate buffer. Six tablets were selected from each formulation and placed in each vessel. Ten milliliters of sample were withdrawn at 1 h interval for 10 h. The sample was then filtered and analyzed for ondensetron HCl spectrophotometrically at 310 nm.

### *in vitro* permeation studies:

Due to comparable water permeability and morphological similarities with human buccal mucosa, porcine buccal mucosa can be used for evaluation of drug rmeability[32,33]. *in vitro* permeation of ondansetron HCl from tablets through the excised porcine buccal membrane was studied[5,30]. Porcine buccal tissue of domestic pigs was obtained from slaughter house and stored in phosphate buffer pH 6.8 at 4^°^. The membrane was mounted over Franz diffusion cell and tablet placed on the membrane and compartments were clamped together. The donor and receptor compartment was filled with buffer of 6.8 and 7.4, respectively at 37^°^. One millilitre sample was withdrawn from receptor compartment at predetermined time and estimated UV spectrophotometerically[34,35].

### Effect of pH modifiers:

The pH specificity of the drug or the formulation may often affect the controlled release profile. Weakly basic drugs with pH-dependent solubility can experience problems on release from controlled release dosage forms in oral cavity. However, this effect is dependent on pKa of the drug and related to pH of the surrounding fluids. Therefore an attempt was made to study the effect of an organic acid and base on the release and permeability of buccal adhesive ondansetron tablets.

On the basis of microenvironment pH, *in vitro* drug release and *in vitro* bioadhesive strength, formulation (C:SA:G, 2:0.5:6.5) was selected for evaluating the effect of pH modifiers on formulation characteristics. Citric acid and sodium bicarbonate sieved (Sieve No. 200) and incorporated in concentrations 1-5% w/w mixed intimately and compressed. These prepared tablets were then evaluated for microenvironment pH, bioadhesive strength, water uptake, *in vitro* release and permeation.

Statistical analysis of the data obtained from the studies was carried out with either one way analysis of variance (ANOVA) and post hoc Dunnett test or two way ANOVA followed by post hoc Bonferroni comparisons.

## RESULTS AND DISCUSSION

Tablets of ondansetron HCl prepared with different polymer combinations were evaluated for friability, drug content, hardness, disintegration time, thickness, weight uniformity and their values complied with pharmacopoeial limit ranging from 0.12 to 0.40%, 7.25± 0.40 to 8.06± 0.32 mg/tablet, 5.00± 0.50 to 7.33± 0.76 kg/cm^2^, 130 to 170 min., 5.0 to 5.5 mm, 96.50± 3.73 to 100.10± 1.94 g, respectively. Hardness of buccal adhesive tablets of ondensetron HCl prepared by using different polymer proportions of C, SA and G in combination was found to increase with increase in the concentration of all three polymers but the effect of polymer concentration on hardness was found to be more pronounced with gelatin (5.50± 0.50 to 7.33± 0.76 kg/cm^2^; F=3.38, *P*=0.053) and least affected by carbopol-934 (5.50± 0.50 to 5.65± 0.29 kg/cm^2^; F=1.308, *P*=0.331) (Table 1).

**TABLE 1:** EVALUATION OF MUCOADHESIVE TABLETS OF ONDENSETRON HYDROCHLORIDE WITH DIFFERENT POLYMERIC RATIO

Formulation (C: SA: G)	Hardness (Kg/cm2) (Mean± SD, n=3)	Friability (%) (Mean± SD, n=3)	Disintegration time (h.min)	Microenviron-mental pH (Mean±SD, n=3)	Bioadhesion (Mean±SD, n=3)
1:1:1	5.50±0.50	0.12±0.01	2.20	4.99±0.11	23.28±0.86
1:2:1	5.85±0.76	0.35±0.011	2.10	5.14±0.16	22.39±1.78
1:3:1	6.35±0.76	0.23±0.020	2.30	5.21±0.24	21.97±1.48
1:141	7.00±1.00	0.25±0.015	2.20	5.34±0.22	21.54±0.98
1:5:1	5.15±0.29	0.30±0.005	2.40	5.39±0.15	21.25±1.16
2:1:1	5.00±0.50	0.25±0.015	2.30	4.14±0.42*	28.81±0.28*
3:1:1	5.65±0.29	0.20±0.015	2.10	4.05±0.47*	30.64±1.50*
4:1:1	5.00±0.50	0.31±0.036	2.40	3.98±0.53*	31.10±0.21*
5:1:1	5.50±0.50	0.21±0.02	2.50	3.91±0.26	31.53±1.63*
1:1:2	6.33±0.58*	0.24±0.017	2.25	5.39±0.24	23.06±2.07
1:1:3	7.03±0.50	0.26±0.005	2.30	5.45±0.25	22.98±2.18
1:1:4	7.33±0.76	0.25±0.005	2.40	5.75±0.18*	22.75±0.39
1:1:5	7.03±1.00	0.40±0.20	2.45	5.84±0.25*	22.46±0.75

* Evaluation of mucoadhesive tablets of ondensetron hydrochloride prepared with carbopol-934 (C), sodium alginate (SA) and gelatin (G) in different ratio (C:SA:G) (*- *P*<0.05, when compared with control formulation-1:1:1; one way ANOVA followed by Dunnett test).

Bioadhesion and microenvironmental pH are determinant of the performance and acceptability of buccal adhesive tablet. Polymers with high bioadhesion used in the formulation often result in decreased microenvironmental pH and therefore cause less irritation. Hence in the present study the blends of three different polymers were used to have optimum bioadhesion and microenvironmental pH as well. Microenvironment pH determines the drug release and is determinant of buccal irritation. The mean microenvironment pH for the formulation containing value C:SA:G (1:1:1) was 4.99± 0.1. Buccal transmucosal tablets prepared with different proportions of all three polymers for acceptable microenvironmental pH with optimum bioadhesive strength.

The release of the drug exhibiting pH-dependent solubility is largely dependent upon the microenvironment pH[24] and release of weakly basic compound has been enhanced by incorporating adipic acid and fumaric acid[16,18,21,25]. Microenvironment pH increased with increase in sodium alginate C:SA:G (1:1:1 to 1:5:1) and gelatin (1:1:1 to 1:1:5) from 4.99± 0.11 to 5.39±0.15 (F=2.313, *P*=0.1286) and 4.99±0.11 to 5.84±0.25 (F=7.456, *P*=0.0047), respectively (Table 1). Sodium alginate and gelatin is commonly used as antacid in the treatment of esophageal reflux[36] and the basic nature of these polymers contributes in the increase pH with increase in their concentration. However increased carbopol concentration C:SA:G (1:1:1 to 5:1:1) have been found to decrease the microenvironment pH (4.99± 0.11 to 3.91± 0.26, F=3.868, *P*=0.0377) (Table 1) which may result into increased irritation and this results are in well agreement with earlier reports[37]. It is likely that acidic groups present with carbopol might have decreased the microenvironmental pH. Tablets prepared with higher proportion of gelatin C:SA:G (1:1:1 to1:1:5) produce desired pH values and therefore it may cause less irritation to buccal mucosa (F=7.456, *P*=0.0047).

The mean bioadhesive strength for formulation containing C:SA:G (1:1:1) was 23.28± 0.86 as depicted in Table 1. Increase in the content of carbopol-934 concentration (1:1:1 to 5:1:1) increases the bioadhesive strength from 23.28± 0.86 to 31.53± 1.63 (F=30.05, *P*<;0.05), whereas increased sodium alginate concentration from 1:1:1 to 1:5:1 decreases bioadhesive strength from 23.28±0.86 to 21.25±1.16 (F=1.128, *P*=0.3968). Increased proportion of gelatin from 1:1:1 to 1:1:5 seemed to have significant effect on bioadhesive strength (F=0.140, *P*=0.963). Formation of secondary mucoadhesive bonds with mucin because of rapid swelling and interpenetration of the polymer chains in the interfacial region is responsible for greater bioadhesion by carbopol-934, while other polymers undergo only superficial bioadhesion[38] and gelatin, a non-ionic polymer relative to carbopol-943, being an anionic polymer shows better bioadhesion[37].

**Fig. 1:**
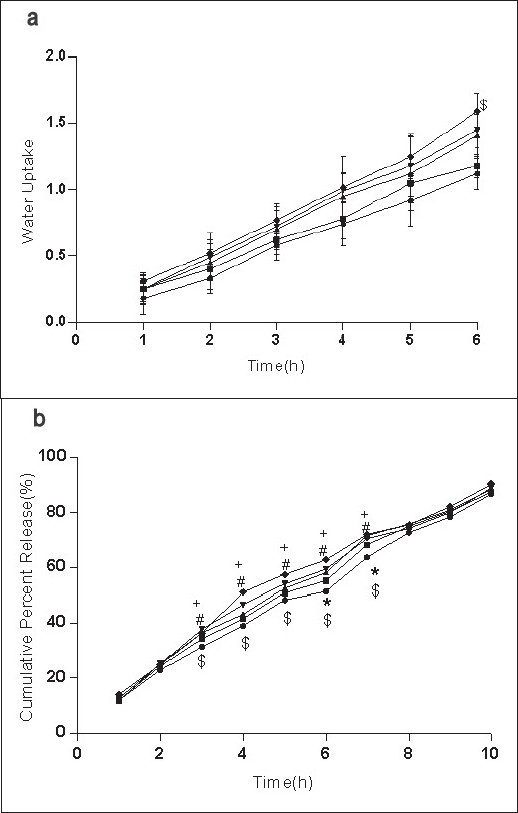
Effect of SA on water uptake and release of Ondansetron HCl from mucoadhesive formulations Effect of carbopol-934 (C), sodium alginate (SA) and gelatin (G) in 1:1:1 –●–), 1:2:1* (–■–), 1:3:1* (–▲–), 1:4:1* (–▼–), 1:5:1* (–♦–) (C:SA:G) on (a) water uptake and (b) cumulative *in vitro* release of ondansetron HCl from buccal mucoadhesive tablets. *P<0.05 when compared with control formulation 2:0.5:6.5; two way ANOVA followed by post hoc Bonferroni test

**Fig. 2:**
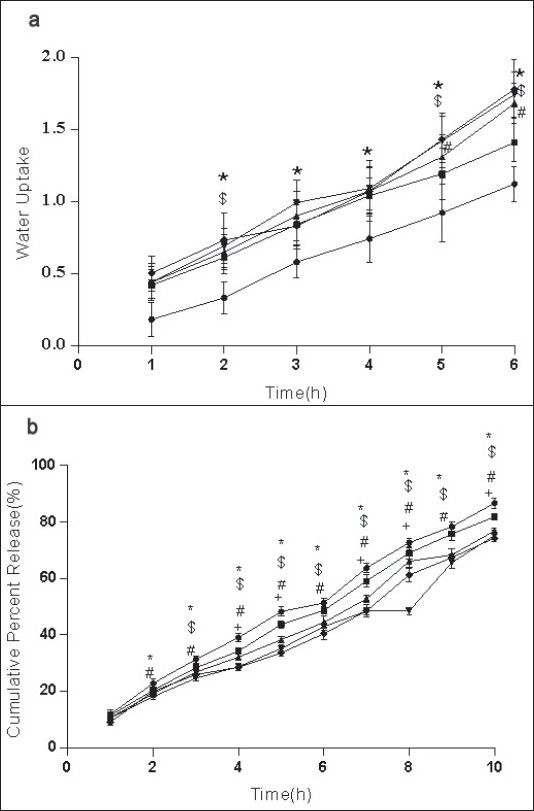
Effect of C on water uptake and release of Ondansetron HCl from mucoadhesive formulation Effect of carbopol-934 (C), sodium alginate (SA) and gelatin (G) in 1:1:1 (–●–), 2:1:1* (–■–), 3:1:1* (–▲–), 4:1:1* (–▼–), 5:1:1* (–♦–) (C:SA:G) on (a) water uptake and (b) cumulative *in vitro* release of ondansetron HCl from buccal mucoadhesive tablets. *P<0.05 when compared with control formulation 2:0.5:6.5; two way ANOVA followed by post hoc Bonferroni test

For the formulation (C:SA:G; 1:1:1) the water uptake value was 1.2±0.12 after 6 h (fig. 1a,2a,3a). Water uptake was 1.41±0.13 to 1.78±0.20 g (fig. 2a) that found to be increased with increasing the concentration of carbopol-934 (F=0.989, *P*=0.431). With increased sodium alginate concentration the water uptake enhanced insignificantly (F=0.389, *P*=0.8145) (fig. 1a) but the effect was less as compare to carbopol-934 and this may be due to more hydrophilic nature of carbopol-934[39].

**Fig. 3:**
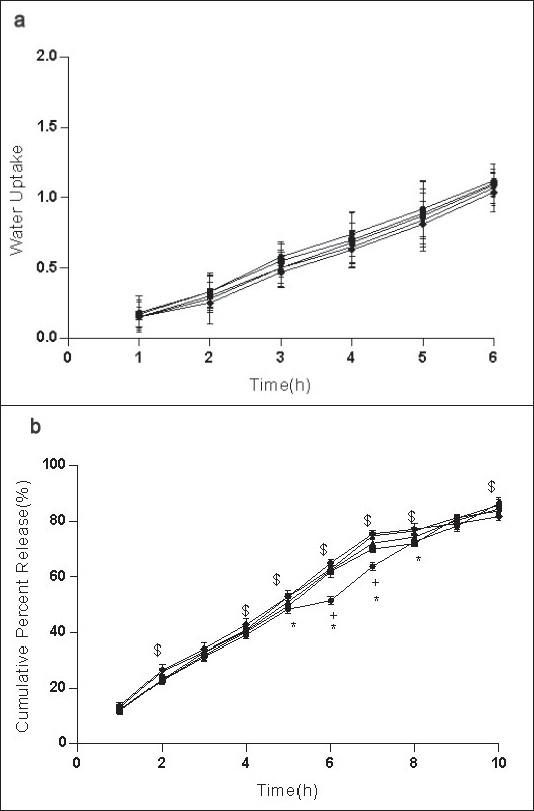
Effect of G on water uptake and release of ondansetron HCl from mucoadhesive formulation Effect of carbopol-934 (C), sodium alginate (SA) and gelatin (G) in 1:1:1 (–●–), 1:1:2* (–■–), 1:1:3* (–▲–), 1:1:4* (–▼–), 1:1:5* (–♦–) (C:SA:G) on (a) water uptake and (b) cumulative in vitro release of ondansetron HCl from buccal mucoadhesive tablets. *P<0.05 when compared with control formulation 2:0.5:6.5; two way ANOVA followed by post hoc Bonferroni test

Ondansetron HCl was found to release more rapidly from the formulation with lower concentrations of carbopol-934 (fig. 2a) as compare to formulation with similar contribution of other polymers. An increase in the polymer concentration increases viscosity of the gel as well as forms gel layer which increases the diffusional path. This could cause a decrease in the effective diffusion coefficient of the drug and therefore a reduction in release rate of drug[39]. Increased gelatin resulted in decreased ondansetron release (fig. 3b). The release of ondansetron could be prolonged and controlled by carbopol and gelatin in a concentration dependent manner. Similar results are reported by Mohammed and Khedr[36].

Based on the above mentioned studies on microenvironment pH, *in vitro* bioadhesion, water uptake and *in vitro* release, tablet formulation containing C:SA:G in proportions 2:0.5:6.5 of ondansetron HCl were prepared and used to investigate the influence of citric acid and sodium bicarbonate on formulation characteristics.

The mean microenvironment pH values after 2 h decreased (Table 2) with the increasing concentration of citric acid from 1% to 5% w/w (F=28.26, *P*<0.05) and increased with the increasing sodium bicarbonate concentration (F=19.31, *P*<0.05). This increase in the microenvironment pH could be attributed to increase sodium bicarbonate concentration which being a base has pH of 8.3 (freshly prepared 0.1 M aqueous solution at 25°)[22]. Earlier reports are available on the incorporation of acidic pH modifiers enhanced the drug release by creating a more acidic microenvironment, increasing the solubility and dissolution rates. The enhanced release of weakly basic drugs by incorporated pH modifiers occurs mainly through modulation of the pH[20].

**TABLE 2:** EFFECT OF CITRIC ACID AND SODIUM BICARBONATE (5-20% W/W) ON MICROENVIRONMENTAL pH AND BIOADHESIVE STRENGTH

Formulation	Concentration	Microenvironment pH Mean±SD (n=3)	Bioadhesive strength Mean±SD (n=3)
Control formulation	0%w/w	5.48±0.28	24.86±2.09
Citric acid	1%w/w	4.87±0.27*	24.67±0.31
	2%w/w	4.74±0.20*	24.47±0.88
	3%w/w	4.61±0.10*	24.41±1.14
	4%w/w	4.48±0.08*	24.53±1.44
	5%w/w	4.37±0.08*	24.24±0.72
Sodium bicarbonate	1%w/w	5.72±0.08	23.99±0.92
	2%w/w	5.92±0.16*	23.32±0.79
	3%w/w	6.04±0.21*	23.33±0.71
	4%w/w	6.23±0.10*	22.12±3.56
	5%w/w	6.37±0.16*	22.19±3.62

* Effect of citric acid (CA) and sodium bicarbonate (SB) on microenvironment pH and bioadhesive strength of ondansetron HCl buccal mucoadhesive tablets formulation containing carbopol-934, sodium alginate and gelatin (C: SA:G) in 2:0.5:6.5 proportion (*- *P*<0.001 when compared with control formulation; one way ANOVA followed by Dunnett test)

The mean bioadhesive strength values (Table 2) were not significantly affected by citric acid (F=0.1855, *P*=0.9626) whereas sodium bicarbonate containing formulations showed a decrease in bioadhesion with increasing concentrations (F=1.768, *P*=0.1940).

**Fig. 4:**
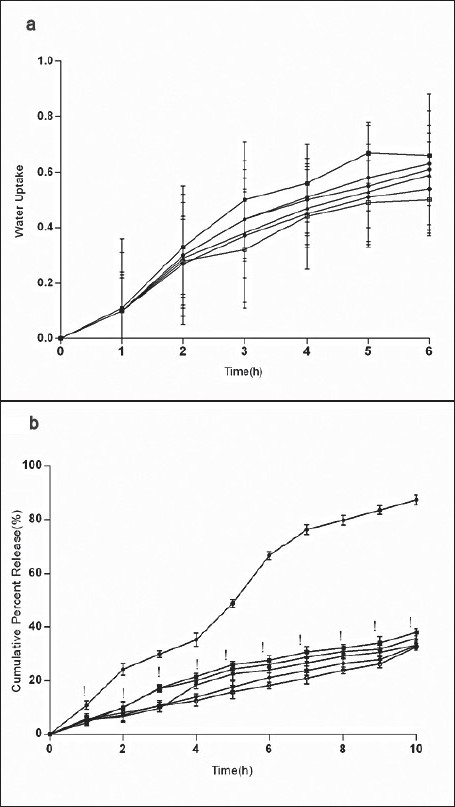
Effect of CA on water uptake and release of ondansetron HCl Effect of various concentrations of citric acid (CA) 0% (–●–), 1%* (–■–), 2 %* (–▲–), 3%* (–▼–), 4%* (–♦–) and 5 %* (–○–) on (a) water uptake and (b) *in vitro* release of ondansetron HCl from buccal mucoadhesive formulation containing carbopol-934 (c), sodium alginate (SA) and gelatin (G) in 2:0.5:6.5 proportions. *P<0.05 when compared with control formulation 2:0.5:6.5; two way ANOVA followed by post hoc Bonferroni test

**Fig. 5:**
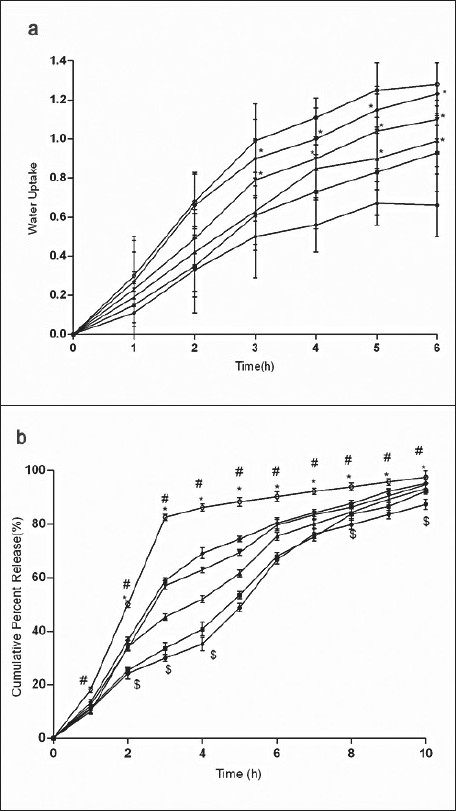
Effect of SB on water uptake and cumulative percent release ondansetron from Effect of sodium bicarbonate (SB) at 0% (–●–), 5% (–■–), 10% (–▲–), 15 % (–▼–) and 20% (–♦–) on (a) water uptake and (b) *in vitro* release of ondansetron HCl from buccal mucoadhesive formulation containing carbopol-934 (c), sodium alginate (SA) and gelatin (G) in 2:0.5:6.5 proportions. *P<0.05 when compared with control formulation 2:0.5:6.5; two way ANOVA followed by post hoc Bonferroni test

For citric acid formulations, the mean water uptake values after 6 h decreased (F=1.047, *P*=0.3958) with the increasing concentration (1-5%w/w, fig. 4a) whereas with sodium bicarbonate formulations the mean water uptake increased (F=18.94, *P*<0.05) with the increasing concentration (1-5%w/w, fig. 5a), respectively. Most of the frequently used pH modifiers are more soluble at higher pH as compared to most basic drug compounds. The pH modifiers diffuse out more rapidly as compared to drug and therefore it is likely that the effect on pH within and in the interface of the dosage form may be decreased[20]. It is probable that increase water uptake with sodium bicarbonate might show decreased bioadhesion.

*in vitro* release of ondansetron HCl decreased significantly from citric acid (fig. 4b) containing formulation (C:SA:G; 2:0.5:6.5) (F=1989, *P*<0.05) whereas increased with sodium bicarbonate (fig. 5b) in concentration dependant manner (F=942.5, *P*<0.05). Release effect was in accordance with the water uptake. Higher water uptake by sodium bicarbonate containing formulation may be a key determinant controlling the release of weakly basic ondansetron HCl.

**Fig. 6:**
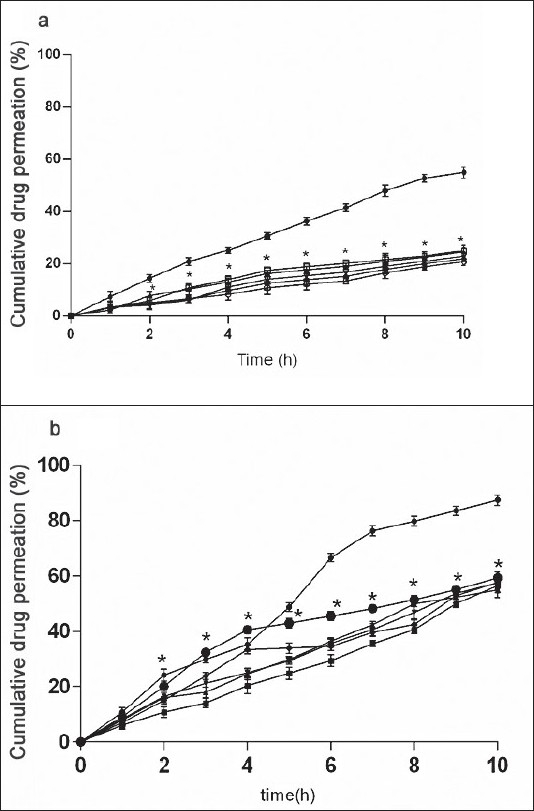
Effect of CA and SB on cumulative drug permeation of mucoadhesive formulation Effect of different concentrations, 0% (–●–), 5% (–■–), 10% (–▲–), 15 % (–▼–) and 20% (–♦–) of (a) citric acid (CA) (b) and sodium carbonate (SB) ‘B’ on *in vitro* permeation of ondensetron HCl from buccal mucoadhesive tablet formulation containing carbopol-934 (c), sodium alginate (SA) and gelatin (G) in 2:0.5:6.5 proportions. *P*0.05 when compared with control formulation 2:0.5:6.5; two way ANOVA followed by post hoc Bonferroni test)

The drug permeation was slow and steady and 54.93±2.23% of ondansetron could permeate through the buccal membrane in 10 h with a average flux of 112.87 μg/h/cm^2^ from control formulation (C:SA:G; 2:0.5:6.5). Drug permeation was reduced to 20.68±1.42% and flux of 40.96 μg/h/cm^2^ from citric acid containing formulation and no significant effect of sodium bicarbonate was observed on permeation (59.33±2.15%) and flux of 121.88 μg/h/cm^2^ of ondansetron from tablets. Drug permeation increases with increased unionized form through biological membrane. Ondansetron, a weakly basic drug is labile for ionization by citric acid and hence the significant reduction in permeation (fig. 6a) through porcine buccal mucosa might have observed. Sodium bicarbonate increases the permeation of ondansetron HCl but to a less significant level (fig. 6b). The insignificant effect observed with sodium bicarbonate may be due to the lesser concentration used in the formulation and for pronounced effect on ionization of ondansetron HCl higher concentration of sodium bicarbonate might be required.

Increasing sodium bicarbonate concentration increases the micorenvironment pH and water uptake and decreases the bioadhesive strength whereas increased carbopol-934 relative to sodium alginate and gelatin decreases the microenvironment pH and increase bioadhesive strength and water uptake. Increased gelatin lead to increased microenvironment pH to more satisfactory levels and decreased bioadhesive strength and water uptake.

Addition of citric acid to the formulation (C:SA:G; 2:0.5:6.5) decreases microenvironment pH, water uptake and drug permeation in a concentration-dependent manner. Increased sodium bicarbonate was found to increase the microenvironment pH, water uptake, drug permeation but decreases the bioadhesive strength. The buccal delivery system of ondansetron HCl formulated in this study was feasible for buccal administration, and use of pH modifier can be used for controlled and desired release profile.

## References

[1] Devarajan PV, Adani MH, Jain NK (2004). Oral Transmucosal Drug Delivery. Controlled Drug Delivery System.

[2] Shojaei AH (1996). Buccal mucosa as a route for systemic drug delivery: A Review. J Pharm Sci.

[3] Verma MV, Kaushal AM, Garg S (2005). Influence of micro-environmental pH on the gel layer behaviour and release of a basic drug from various hydrophilic matrices. J Control Release.

[4] Gutsche S, Krause M, Kran H (2008). Strategies to overcome pH-dependent solubility of weakly basic drugs by using different types of alginates. Drug Dev Ind Pharm.

[5] Squier CA, Wertz PW, Rathbone MJ (1996). Structure and function of the oral mucosa and implications for drug delivery. Oral Mucosal Drug Delivery.

[6] Lehman K, Sellasie IG (1994). Coating of multiparticulate using polymeric solution: Formulation and Process Consideration. Drugs and Pharmaceutical Sciences, Multiparticulate oral drug delivery.

[7] Follonier N, Doelker E, Cole ET (1995). Various ways of modulating the release of diltiazem hydrochloride from hot-melt extruded sustained release pellets prepared using polymeric materials. J Control Rel.

[8] McGinity JW, O’Donnell PB (1997). Preparation of microspheres by solvent evaporation techniques. Adv Drug Deliv Rev.

[9] Savage GV, Rhodes CT (1995). Sustained release coating of solid dosage forms: A historical review. Drug Dev Ind Pharm.

[10] Bose S, Bogner RH (2007). Solventless photocurable film coating: Evaluation of drug release, mechanical strength and photostability. AAPS PharmSciTech.

[11] Delargy AM, Timmins P, Minhom C, Howard JR (1989). The optimization of a pH-independent matrix for the controlled release of drug materials using *in vitro* modeling. Proc Int Symp Control Rel Bioact Mater.

[12] Al-Sayed-Omar O, Johnston A, Turner P (1987). Influence of pH on the buccal absorption of morphine sulphate and its major metabolite, morphine-3-glucuronide. J Pharm Pharmacol.

[13] Roy S, Pal K, Anis A, Pramanik K, Prabhakar B (2009). Polymers in mucoadhesive drug delivery system: A Brief Note. Designed Monomers Polymers.

[14] Nair MK, Chetty DJ, Ho H, Chien YW (1997). Biomembrane permeation of nicotine: Mechanistic studies with porcine mucosae and skin. J Pharm Sci.

[15] Thoma K, Ziegler I (1998). The pH-independent release of fenoldopam from pellets with insoluble film coats. Eur J Pharm Biopharm.

[16] Streubel A, Siepmann J, Dashevsky A, Bodmeier R (2000). pH-independent release of a weakly basic drug from water-insoluble and water-soluble matrix tablets. J Control Release.

[17] Espinoza R, Hong E, Villafuerte L (2000). Influence of admixed citric acid on the release profile of pelanserin hydrochloride from HPMC matrix tablets. Int J Pharm.

[18] Nie S, Pan W, Li X (2004). The effect of citric acid added to hydroxypropyl methylcellulose (HPMC) matrix tablets on the release profile of vinpocetine. Drug Dev Ind Pharm.

[19] Nykanen P, Slempaa M, Aaltonen L, Jurjenson H, Marvola M (2001). Citric acid as an excipient in multiple unit enteric coated tablets for targeting drugs to the colon. Int J Pharm.

[20] Siepe S, Lueckel B, Kramer A, Ries A, Gurny R (2006). Strategies for the design of hydrophilic matrix tablets with controlled microenvironmental pH. Int J Pharm.

[21] Burnside BA, Chang RK, Guo X (2001). Sustained release pharmaceutical dosage forms with minimized pH dependent dissolution profi les. US Patent No. 6,287,599.

[22] Budavari S (1996). The Merck Index- An Encyclopedia of Chemicals, Drugs and Biological.

[23] Reilly WJ, Gennaro AR (1998). Pharmaceutical Necessities. The Science and Practice of Pharmacy.

[24] Rao VM, Engh K, Qiu YH (2003). Design of pH-independent controlled release matrix tablets for acidic drugs. Int J Pharm.

[25] Gabr KE (1992). Effect of organic acids on the release patterns of weakly basic drugs from inert sustained release matrix tablets. Eur J Pharm Biopharm.

[26] Bottenberg P, Cleymet R, Muynek CD, Remen JP, Coomans D, Slop D (1991). Development and testing of bioadhesive, fluoride-containing slow-release tablets for oral use. J Pharm Pharmacol.

[27] Gupta A, Garg S, Khar RK (1992). Measurement of bioadhesive strength of muco-adhesive buccal tablets: design of an in-vitro assembly. Indian Drugs.

[28] Parodi P, Russo E, Caviglioli G, Caffagi S, Bignardi G (1996). Development and characterization of a buccoadhesive dosage form of oxycodone hydorchloride. Drug Dev Ind Pharm.

[29] Agarwal V, Mishra B (1999). Design, development and biopharmaceutical properties of buccoadhesive compact of pentazocine. Drug Dev Ind Pharm.

[30] Tabak LA, Levine MJ, Mandel ID, Ellison SA (1982). Role of salivary mucins in the protection of the oral cavity. J Oral Pathol.

[31] Mannivanan R, Balasubramaniam A, Anand Prem DC, Sandeep G, Rajkumar N (2008). Formulation and *in vitro* evaluation of mucoadhesive buccal tablets of diltiazem hydrochloride. Res J Pharm Tech.

[32] Lesch CA, Squier CA, Cruchley A, Williams DM, Speight P (1989). The permeability of human oral mucosa and skin to water. J Dent Res.

[33] Hao Z, Robinson JR (1996). *in vitro* methods for measuring permeability of the oral mucosa. Drugs Pharm Sci.

[34] Wertz PW, Squier CA (1991). Cellular and molecular basis of barrier function in oral epithelium. Crit Rev Ther Drug Carrier Syst.

[35] Gerson SJ, Harris RR, Meyer J, Squier CA, Gerson SJ (1984). The Structure and Function of Oral Mucosa.

[36] Mohammed FA, Khedr H (2003). Preparation and *in vitro/in vivo* evaluation of the buccal bioadhesive properties of slow-release tablets containing miconazole nitrate. Drug Dev Ind Pharm.

[37] Nafee NA, Ismail FA, Boraie NA, Mortada LM (2004). Mucoadhesive delivery systems: Evaluation of Mucoadhesive polymers for buccal tablet formulation. Drug Dev Ind Pharm.

[38] Senel S, Duchene D, Hincal AA, Capan Y, Ponchel G (1998). *in vitro* studies on enhancing effect of sodium glycocholate on transbuccal permeation of Morphine hydrochloride. J Control Release.

[39] Siepe S, Herrmann W, Borchert HH, Leuckel B, Kramer A, Ries A (2006). Microenvironmental pH and microviscosity inside pH-controlled matrix tablets: An EPR imaging study. J Control Release.

